# Accuracy of aortic pulse wave velocity assessment with velocity-encoded MRI: validation in patients with Marfan syndrome

**DOI:** 10.1186/1532-429X-13-S1-O71

**Published:** 2011-02-02

**Authors:** Eleanore S Kröner, Rob J van der Geest, Arthur J Scholte, Pieter J van den Boogaard, Dennis Hendriksen, Lucia J Kroft, Maarten Groenink, Teodora Radonic, Jeroen J Bax, Albert de Roos, Johan H Reiber, Jos J Westenberg

**Affiliations:** 1 Leiden University Medical Center, Leiden, Netherlands; 2 Academic Medical Center, Amsterdam, Netherlands

## Introduction

The leading cause of premature death in patients with Marfan syndrome (MFS) is aortic dissection and subsequent rupture after progressive aorta dilatation due to increased wall stiffening. Pulse Wave Velocity (PWV), defined as the systolic flow velocity wave front propagation speed through the aorta, is a marker of wall stiffness with proven prognostic value in MFS. Recently it was demonstrated that, although time-consuming, PWV-assessment from two-directional in-plane velocity-encoded MRI covering the whole aorta in three parallel oblique-sagittal slices is the most accurate approach (1). 2-slice free-breathing through-plane velocity-encoded MRI at three locations perpendicular to the aorta (2) is traditionally used for PWV-estimation. Accelerated multi-slice acquisition with breath-holding and more-densed sampling along the aorta might improve accuracy.

## Purpose

To examine accuracy of PWV-assessment from 2-slice free-breathing and 4-slice breath-held through-plane velocity-encoded MRI in MFS.

## Methods

**Figure 1:**
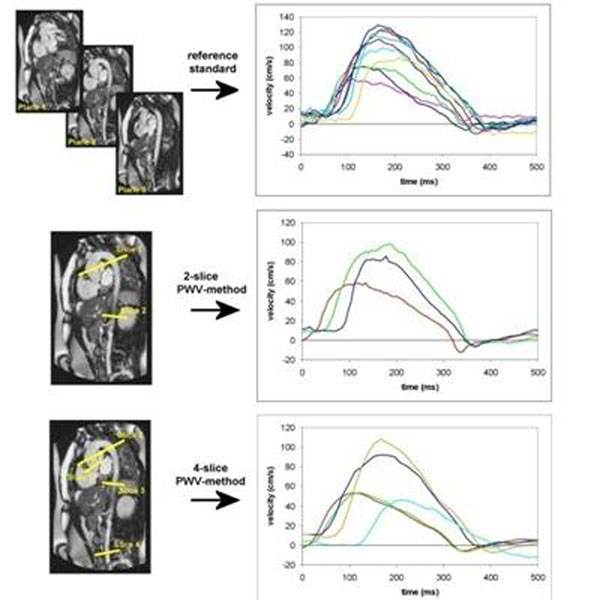
Methods for PWV-assessment: two-directional in-plane velocity-encoded MRI (reference standard), 2-slice free-breathing one-directional through-plane velocity-encoded MRI, 4-slice breath-held one-directional through-plane velocity-encoded MRI. PWV is determined from the systolic velocity wave front propagation.

Eighteen MFS patients (9 men, mean age=33±12years) were included. PWV-assessment was performed on 1.5T MRI (Philips Medical Systems, Best, the Netherlands). Three methods were evaluated; 1) reference standard: two-directional in-plane velocity-encoding with high temporal resolution=9ms in three parallel oblique-sagittal slices covering the whole aorta, sampling PWV at 200 equidistant points along the aorta; 2) traditional 2-slice method: two free-breathing through-plane velocity-encoded acquisitions with high temporal resolution=10ms at the proximal aorta transecting ascending and proximal descending aorta and at the abdominal aorta; 3) accelerated 4-slice method: four breath-held velocity-encoded acquisitions with inferior temporal resolution=23ms and echo-planar-imaging factor 11 at five locations (aortic valve, ascending and proximal descending aorta, diaphragm and above the bifurcation) (Figure 1). Aortic PWV-assessment was compared to the reference standard.

## Results

**Figure 2:**
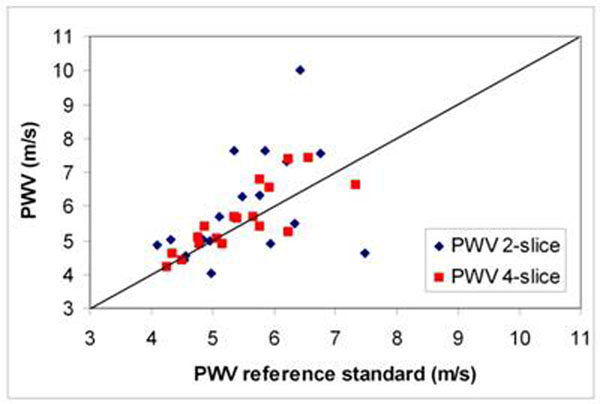
Correlation for 2-slice and 4-slice PWV-assessment with the reference standard.

MRI-results are presented in Table 1 and Figure 2. Despite inferior temporal resolution, 4-slice PWV-method showed stronger correlation with the reference standard and less error and variation compared to 2-slice method. Of note, 4-slice method also resulted in 75% scan time reduction.

**Table 1:** MRI-results

	PWV 2-slice method	PWV 4-slice method
Pearson r	0.47	0.82
Mean difference ± standard deviation (m/s)	0.40 ± 1.40	0.18 ± 0.56
p-value t-test	0.24	0.19
Mean unsigned error	18%	8%
Coefficient of variation	25%	10%
Mean total scan time	8 minutes	2 minutes

## Conclusions

4-slice breath-held through-plane velocity-encoded MRI at five locations perpendicular to the aorta improves PWV-assessment versus traditionally-used 2-slice free-breathing velocity-encoded MRI, despite inferior temporal resolution.
